# Neuromagnetic representation of melodic contour processing in human auditory cortex

**DOI:** 10.3389/fnhum.2022.909159

**Published:** 2022-10-26

**Authors:** Sabrina Taddeo, Martin Schulz, Martin Andermann, André Rupp

**Affiliations:** ^1^Department of Otolaryngology, Head and Neck Surgery, University Medical Center of Tübingen, Tübingen, Germany; ^2^Section of Biomagnetism, Department of Neurology, Heidelberg University Hospital, Heidelberg, Germany

**Keywords:** pitch, auditory cortex, source analysis, MEG (magnetoencephalography), melodic contours

## Abstract

The pattern of ups and downs in a sequence with varying pitch can be heard as a melodic contour. Contrary to single pitch, the neural representation of melodic contour information in the auditory cortex is rarely investigated, and it is not clear whether the processing entails a hemispheric asymmetry. The present magnetoencephalography study assessed the neuromagnetic responses of *N* = 18 normal-hearing adults to four-note sequences with fixed vs. varying pitch that were presented either monaurally or diotically; data were analyzed using minimum-norm reconstructions. The first note of the sequences elicited prominent transient activity in posterior auditory regions (Planum temporale), especially contralateral to the ear of entry. In contrast, the response to the subsequent notes originated from more anterior areas (Planum polare) and was larger for melodic contours than for fixed pitch sequences, independent from the ear of entry and without hemispheric asymmetry. Together, the results point to a gradient in the early cortical processing of melodic contours, both in spatial and functional terms, where posterior auditory activity reflects the onset of a pitch sequence and anterior activity reflects its subsequent notes, including the difference between sequences with fixed pitch and melodic contours.

## Introduction

Pitch has received much attention in auditory research, including both invasive and non-invasive endeavors to study the underlying neural processing in human listeners (Gutschalk et al., 2002, 2004; Krumbholz et al., 2003; Ritter et al., 2005, 2007; Seither-Preisler et al., 2006; Schönwiesner and Zatorre, 2008; Andermann et al., 2014, 2017, 2021; Gander et al., 2019; Kim et al., 2022). When the pitches in a sound sequence vary, we can perceive this pattern of ups and downs as a melodic contour (Dowling, 1978), based on a musical scale framework that is largely culture-specific (Dowling, 2010). The “meaningful” information (Roederer, 2000, p. 6) conveyed by a melody can already be extracted from very brief contours like jingles, ringtones and even alarms (Gillard and Schutz, 2016); however, despite their omnipresence, the processing of melodic contours in human auditory cortex is not well understood.

Functional magnetic resonance imaging (fMRI) studies suggest that the cortical representation of melodic contours involves a relatively high level of information processing, in contrast to activation that reflects single pitch (Patterson et al., 2002; Hall and Barker, 2012). Early work comparing sequences with fixed vs. varying pitch observed that the activity related to pitch variation occurs in putative belt and parabelt auditory regions anterior to primary auditory cortex (Griffiths et al., 1998, 2001; Patterson et al., 2002). These studies compared sequences of sounds that elicited no pitch percept with sequences in which all sounds had the same (i.e., fixed) pitch, and with sequences in which pitch varied to produce a short melodic contour. While the contrast of the pooled (i.e., averaged) sequences with a rest condition (without any auditory stimulation) activated virtually the entire auditory cortex bilaterally, the between sequence contrasts revealed a hierarchical order of processing: The contrast of fixed pitch with no pitch sequences elicited responses in the anterolateral Heschl’s gyrus (HG) in both hemispheres, and the contrast of melodic contours and fixed pitch sequences showed activation that extended from the lateral aspect of HG to the Planum polare (PP). This pattern was similar between Griffiths’ positron emission tomography (PET) study and Patterson’s fMRI experiment, but the fMRI results exhibited a somewhat stronger hemispheric asymmetry with more activation on the right side. Although subsequent studies corroborated the assumption of a hierarchical structure (Warren and Griffiths, 2003; Hall and Barker, 2012; Janata, 2015) in the cortical processing of melodic contours, it is still unclear whether these aspects can also be derived in neurophysiological measurements which have a finer temporal resolution.

Existing electro- and magnetoencephalography (EEG/MEG) studies have tackled numerous aspects of melodic contour processing, including a variety of neural response components and elaborated modeling frameworks (e.g., Fujioka et al., 2004; Brattico et al., 2006; Pearce et al., 2010; Quiroga-Martinez et al., 2019, 2020). For example, a recent MEG experiment from our group (Andermann et al., 2021) investigated the cortical correlates of absolute (i.e., tone height) and relative (i.e., shift size and direction) pitch information in sequences with fixed vs. varying pitch. The transient neuromagnetic activity was found to mirror absolute pitch information at sequence onsets and offsets, and relative pitch information within the sequences; notably, fixed pitch sequences elicited much smaller responses than sequences with varying pitch. The study of Andermann et al. (2021) is one of only few who employed a design with equiprobable stimuli, together with source level analyses of the cortical activity (P50m, N100m, P200m, sustained field); in contrast, many other EEG/MEG studies on melodic contour processing applied oddball paradigms and/or focused on components like the mismatch negativity or the P300 wave (e.g., Fujioka et al., 2004; Quiroga-Martinez et al., 2019). On the other hand, Andermann et al. (2021) did not consider a conceptual or anatomical hierarchy of pitch processing, and the octave sequences in their study certainly represent a special case of melodic contours.

The first goal of the current study was to compare the neurophysiological response to short four-note sequences with and without a melodic contour. As in our previous work (Andermann et al., 2021), we used a paradigm with equiprobable stimuli; but the tonal range of the stimulus set was designed such that the melodic contours had a somewhat more realistic (i.e., jingle-like) character. Assuming that melodic contour processing occurs at higher cortical levels (e.g., Patterson et al., 2002; Warren and Griffiths, 2003; Norman-Haignere et al., 2013), we expected that the second, third and fourth note of those contours would elicit enhanced responses in anterior auditory cortex; in contrast, activity in more posterior areas was expected to show no difference between melodic contours and fixed pitch sequences (Gutschalk et al., 2002). In an effort to compare our results with the above-mentioned findings, and to avoid confounders, the temporal structure of the pitch sequences (i.e., rhythm) was kept fixed in the present study, and we solely focused on the aspect of melodic pitch variation within the sequences.

The second goal of our study was to find out whether the postulated right-hemispheric dominance of melodic contour processing at anterior sites (Patterson et al., 2002) would also be visible in neuromagnetic recordings. Generally speaking, contralateral dominance of neural responses to monaural stimulation has been described in both fMRI and EEG/MEG studies, but the overall result pattern remains elusive with respect to the degree of asymmetry at different anatomical and functional processing stages (e.g., Reite et al., 1982; Pantev et al., 1986; Näätänen and Picton, 1987; Gutschalk and Steinmann, 2015). Specifically, the response to melodic stimuli was pronounced in the right auditory cortex in Patterson et al.’s (2002) fMRI study and also in neurophysiological investigations (e.g., Brattico et al., 2006), but other studies did not reveal comparable hemisphere effects (e.g., Fujioka et al., 2004; Janata, 2015; Andermann et al., 2021). In the current experiment, sound sequences were presented either monaurally (i.e., solely to the left or right ear) or diotically, assuming that rightward lateralization in the neural representation of melodic contours (cf. Patterson et al., 2002) should occur independent of the ear of entry, in contrast to the overall onset response.

## Materials and methods

### Participants

Eighteen adult volunteers (6 females, 1 left-handed; mean-age: 32.5 ± 6.8 years) participated after providing written informed consent. The experimental procedures were conducted in accordance with the Declaration of Helsinki, and they were approved by the local ethics committee of the Medical Faculty, University of Heidelberg (S441/2016). None of the listeners reported any history of neurological or hearing disorders. For individual source localization, T1-weighted high-resolution magnetization-prepared rapid gradient echo (MPRAGE) structural MRI data were acquired from the participants using a 3.0 T Siemens Trio scanner (Siemens Medical Systems, Erlangen, Germany).

### Stimuli

**FIGURE 1:**
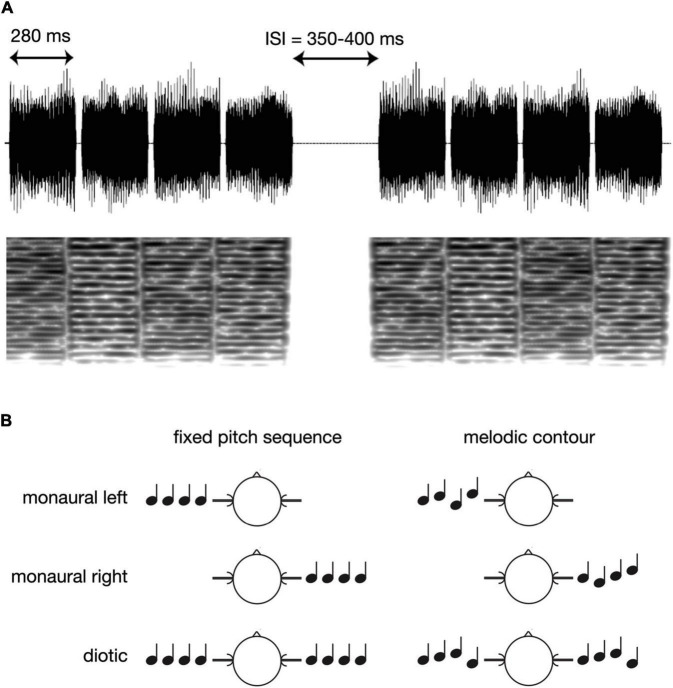
Schematic overview of the experimental paradigm. **(A)** Examples of the IRN sound waveforms in the melodic contour condition, together with the corresponding spectrograms. **(B)** Presentation of fixed pitch sequences vs. melodic contours in the monaural left, monaural right, or diotic condition.

Figure 1 presents a schematic overview of the experimental paradigm. The four-note sequences for the experiment were generated using Matlab 7.1 (The MathWorks, Inc., Natick, MA, USA) at a sampling rate of 48,000 Hz, and with 16 bit resolution. All sounds were based on iterated rippled noise (IRN; Yost, 1996) which is generated by copying a rippled noise and adding it to the original signal with a time delay. The fundamental frequency (*f*_0_) of the resulting pitch corresponds to the reciprocal of the delay, and the percept becomes more salient when more iterations are added. In the current study, we used IRN sounds with eight iterations, and with four different delays: 10.2, 11.45, 12.13, and 13.62 ms (corresponding to the following *f*_0_ values: 98.04, 87.34, 82.44, and 73.42 Hz, i.e., the musical notes G, F, E, and D). The duration of each note was 280 ms, including 10 ms Hanning windows at the onset and offset, and all notes were bandpass filtered between 500 and 4,000 Hz. The single notes were then assembled to short four-note sequences with either fixed or varying *f*_0_, and with 20 ms pause intervals between the single notes. The fixed pitch sequences were balanced such that all four *f*_0_ values occurred equally often whereas sequences with varying *f*_0_ (melodic contours) were based on all possible combinations of the four different *f*_0_ values (cf. Figure 1A). A total of 336 sequences of each type were presented, in pseudo-random order, and the inter-sequence interval varied randomly between 350 and 400 ms. Further, sequence presentation was balanced such that monaural left, monaural right and diotic stimulation occurred equally often (cf. Figure 1B), and also in pseudo-random order. The overall level was set to 70 dB SPL.

### Magnetoencephalography recordings

The neuromagnetic field gradients in response to the four-note sequences were measured using a Neuromag-122 MEG system (Elekta Neuromag Oy, Helsinki, Finland; Ahonen et al., 1993) inside a shielded room (IMEDCO, Hägendorf, Switzerland), and with a sampling rate of 1,000 Hz and a bandpass filter of DC-330 Hz. Stimuli were presented *via* Etymotic Research (ER3) earphones attached to 90 cm plastic tubes and foam earpieces using a 24-bit sound card (RME ADI 8DS AD/DA converter), an attenuator (Tucker-Davis Technologies PA-5) and a headphone buffer (Tucker Davis Technologies HB-7). The position and orientation of the head under the MEG dewar was determined prior to the measurement using four head position indicator coils; coil positions were digitized before the MEG recording using a Polhemus 3D-Space Isotrack2 system, together with the preauricular points, the nasion and 100 surface points around the head. Co-registration of the MEG and MRI data was based on these fiducial and head surface points. During data acquisition, participants watched a silent movie of their own choice; they were instructed to direct their attention to the movie. Off-line analysis was based on the continuous raw data.

### Data analysis and source reconstruction

Data were analyzed with MNE-Python v0.24 (Gramfort et al., 2013, 2014). First, MEG signals were visually inspected and noisy channels were removed. The data were then bandpass filtered with a zero-phase FIR filter from 1 to 30 Hz (zero-double) for all further analyses, and epoched into sweeps of 1.8 s duration with a baseline ranging from –0.1 to 0 s. Automatic artifact rejection was based on the autoreject method (Jas et al., 2017). After averaging the data, the digitized head shape was used to coregister the brain model. DICOM files of the individual MRI T1-weighted images were processed using the recon-all procedure of FreeSurfer v7.1.1 (Dale et al., 1999; Fischl et al., 1999) to generate the cortical surface, boundary element model, and the source spaces. For source space, the ico5 subsampling procedure was used to compute 20,484 vertices on the white surface (the layer between the gray and white matter) which results in a source spacing of about 3.1 mm. In order to visualize the data of the sulci, surfaces were inflated (Dale et al., 1999). The watershed algorithm (Ségonne et al., 2004) was used to create a boundary element model (BEM) of the inner skull surface. For the inverse operator, the dSPM (dynamic statistical parametric mapping) method with a regularization parameter of 0.11 was chosen (Dale et al., 2000). No constraints were applied regarding dipole orientation, and the baseline was used to estimate the noise covariance matrix. The source reconstruction was then projected onto the aparc_sub atlas (Khan et al., 2018; their Figure 1) which includes a high resolution parcellation scheme consisting of 448 cortical labels.

**FIGURE 2:**
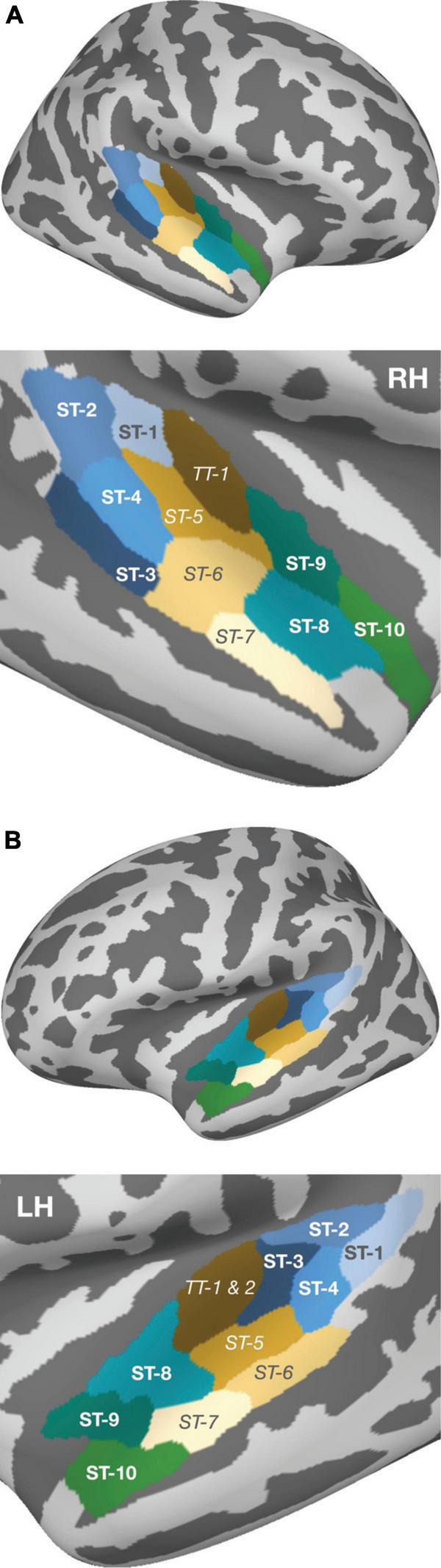
Parcellation of the auditory cortex according to the aparc_sub atlas (Khan et al., 2018), provided by the MNE software, and projected onto the fsaverage reconstruction. Within panels **(A)** (right hemisphere) and **(B)** (left hemisphere), the upper plots depict the whole brain view and the lower plots present an enlarged view of the temporal lobe. Green- and blue-shaded areas indicate the ROIs [i.e., the cortical subregions in PT (blue) and PP (green)] that formed the basis for subsequent source wave extraction. The areas between both ROIs are shown in beige/brown, with their labels in italics; these areas were not used for statistical testing and are illustrated merely to graphically emphasize the spatial separation of the two ROIs that were in the focus of the current study.

Subsequently, a two-stage analysis was conducted to evaluate the spatio-temporal activity in response to the stimulation. In a first step, two symmetric regions of interest (ROIs) were defined, in both hemispheres, to extract the neuromagnetic activity in posterior and anterior areas of the auditory cortex. The posterior ROI was determined based on coordinates reported in the fMRI studies of Barrett and Hall (2006; their Table 3) and Warren and Griffiths (2003; their Table 1). The anatomical areas that most prominently mirrored spatial contrasts in both works were mapped onto the fsaverage brain using Freeview v3 (Dale et al., 1999); they corresponded to superior temporal areas 1–4 in the aparc_sub atlas and roughly covered the posterior portion of Planum temporale (PT; cf. Figure 2). In an analogous manner, the anterior ROI was defined based on the same two works (Warren and Griffiths, 2003; Barrett and Hall, 2006), and additionally on the fMRI study of Patterson et al. (2002) on melody processing; here, superior temporal areas 8–10 were reported as the prominent areas that reflected melodic contour contrasts, roughly covering PP.

In the second step, the pooled dSPM-model source waveforms associated with the above-specified ROIs were extracted from MNE-Python and were exported, separately for every participant, hemisphere, ROI, and experimental condition, to MATLAB for plotting, and to the SAS Studio software 3.8 (Cary, NC, USA) for statistical analysis. In an effort to compensate for any confounding effects of N100m latency, the response magnitudes fed into the analyses were calculated as the mean activity within a 60 ms interval centered around the grand-average N100m peak in each single experimental condition (Figure 1B) and hemisphere. The statistical evaluation was done using repeated measure ANOVAs (GLM procedure in SAS), separately for the onset response (i.e., the response to the first note) and for the pooled within-sequence responses (i.e., the responses to the second, third and fourth note), with Greenhouse–Geisser corrections for sphericity violations. All ANOVAs included EAR OF ENTRY (monaural left vs. monaural right vs. diotic stimulation), SEQUENCE (fixed pitch sequence vs. melodic contour) and HEMISPHERE (left vs. right) as within-subject factors and assessed their respective main effects and interactions. To determine specific contrasts, additional pairwise *t*-tests were performed, with *p*-values corrected for multiple comparisons using the Holm–Bonferroni method (Holm, 1979).

## Results

**FIGURE 3:**
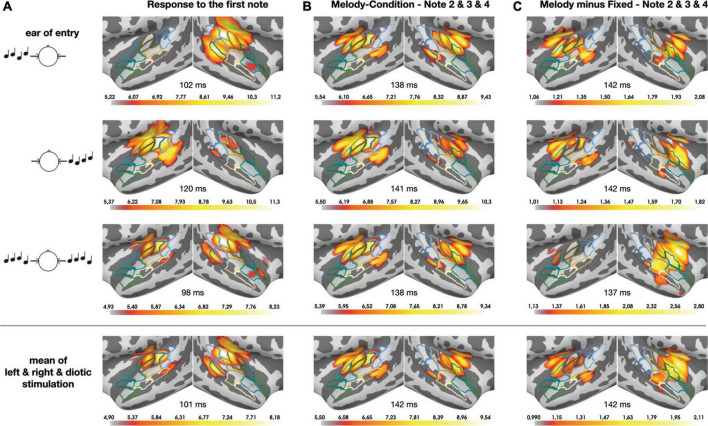
Temporal lobe MNE representation (dSPM) of the neural responses in the monaural left and right (top and 2nd panels) and the diotic (3rd panels) listening conditions, separately for the first note **(A)** and for the pooled second, third, and fourth note **(B)** of the (melodic) sequences, as well as for the pooled within-sequence activity based on the contrast between sequences with fixed vs. varying pitch **(C)**. The bottom panels depict the mean values across the entry-of-ear conditions to illustrate the anterior-posterior shift from panels **(A)** to **(C)** in both hemispheres. All representations cover the time interval of the corresponding N100m waves; small numbers shown between hemispheres indicate the corresponding N100m peak times. Color linings indicate the ROIs in PT (blue) and PP (green), as well as intermediary areas (beige; cf. Figure 2). The color code of the activation results from auto-rescaling within the MNE software, separately for each subplot (minimum: 96th percentile, maximum: 99.5th percentile).

**FIGURE 4:**
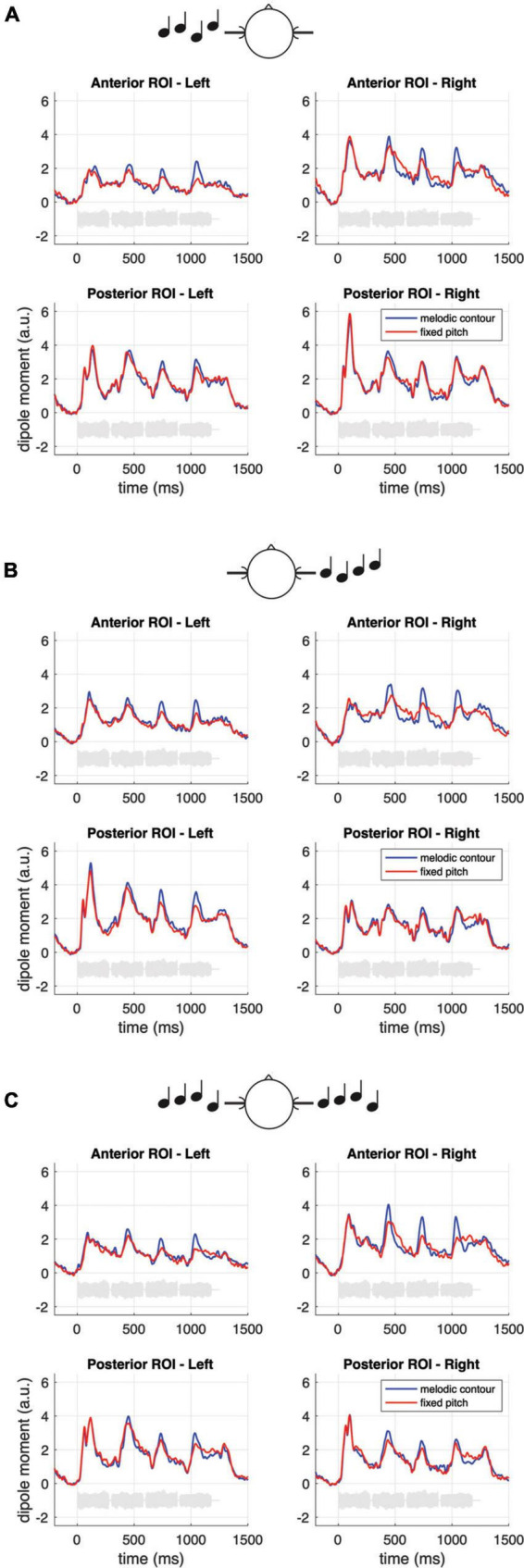
Grand-average source waveforms for melodic contours and fixed pitch sequences, separately for the monaural left and right **(A,B)** and the diotic **(C)** listening conditions, and for the anterior and posterior ROIs. The data represent pooled source waves extracted from the pre-defined ROIs (cf. Materials and methods). The light gray curves in each subplot represent example sound waveforms from the stimulation.

Figure 3 presents the temporal lobe MNE representation of the neural responses in the monaural left and right and the diotic listening conditions, separately for the first note (panel A) and for the pooled second, third and fourth note (panel B) of the sequences, as well as for the pooled within-sequence activity based on the difference (i.e., contrast) between sequences with fixed vs. varying pitch (panel C). The bottom panels of the figure depict mean MNE representation across single conditions; these plots were included in an effort to illustrate overall spatial shifts of activation in both hemispheres. All representations cover the time interval of the N100m wave. The pooled grand-average source waveforms for melodic contours and fixed pitch sequences as derived from the pre-defined ROIs are shown in Figure 4, separately for the monaural left and right (panels A and B) and the diotic (panel C) listening conditions, and for the anterior and posterior ROIs; all relevant *post hoc* tests are summarized in Table 1. Following the onset of the first note, a prominent N100m response evolved in posterior auditory cortex (Figure 3A), in all experimental conditions (bottom panels in Figures 4A–C). As expected, there were no significant main effects of SEQUENCE [*F*(1, 17) = 0.41, *p* = 0.531] or HEMISPHERE [*F*(1, 17) = 0.08, *p* = 0.783]; however, the N100m activity was much larger in the hemisphere contralateral to the ear of entry, as revealed by a highly significant main effect of EAR OF ENTRY [*F*(2, 34) = 7.16, *p* = 0.006^**^] and a highly significant HEMISPHERE * EAR OF ENTRY interaction [*F*(2, 34) = 27.18, *p* < 0.001^***^]. *Post hoc* tests showed that this effect was particularly strong among the monaural conditions, whereas it was not significant when only diotic conditions were considered (cf. Table 1). Surprisingly, the SEQUENCE * EAR OF ENTRY interaction was just significant [*F*(2, 34) = 4.11, *p* = 0.040*]; on the other hand, the SEQUENCE * HEMISPHERE interaction [*F*(1, 17) = 1.35, *p* = 0.262] was not significant, nor was the second-order interaction [*F*(2, 34) = 0.30, *p* = 0.733].

**TABLE 1:** Summary of *post hoc* tests for the reported ANOVAs.

ROI	Note (s)	Sequence (s)	Ear of entry	Contrast	Mean	SD	*t*	*p*	*p*_correct_	90% CI
Posterior	1	Melodic	Monaural left	Right-left	1.31	2.88	1.93	0.036	0.144	[0.13 2.49]
Posterior	1	Melodic	Monaural right	Right-left	–1.85	2.59	–3.03	0.004	**0.020**	[–2.91 –0.79]
Posterior	1	Melodic	Diotic	Right-left	–0.12	2.31	–0.22	0.413	0.826	[–1.07 0.82]
Posterior	1	Fixed	Monaural left	Right-left	1.35	3.06	1.88	0.039	0.144	[0.10 2.61]
Posterior	1	Fixed	Monaural right	Right-left	–1.58	2.11	–3.18	0.003	**0.018**	[–2.44 –0.71]
Posterior	1	Fixed	Diotic	Right-left	–0.05	2.60	–0.08	0.469	0.826	[–1.12 1.02]
Posterior	1	All	Monaural left	Right-left	1.33	2.94	1.92	0.036	0.072	[0.25 5.07]
Posterior	1	All	Monaural right	Right-left	–1.71	2.30	–3.16	0.003	**0.009**	[–5.31 –1.54]
Posterior	1	All	Diotic	Right-left	–0.09	2.43	–0.15	0.442	0.442	[–2.17 1.83]
Anterior	2, 3, and 4	Melodic	Monaural left	Right-left	1.04	1.29	3.41	0.002	**0.010**	[0.51 1.57]
Anterior	2, 3, and 4	Melodic	Monaural right	Right-left	0.96	1.10	2.65	0.008	**0.032**	[0.24 1.14]
Anterior	2, 3, and 4	Melodic	Diotic	Right-left	0.97	1.54	2.67	0.008	**0.032**	[0.34 1.60]
Anterior	2, 3, and 4	Fixed	Monaural left	Right-left	1.06	1.01	4.48	< 0.001	**0.006**	[0.65 1.48]
Anterior	2, 3, and 4	Fixed	Monaural right	Right-left	0.53	0.95	2.40	0.014	**0.028**	[0.15 0.92]
Anterior	2, 3, and 4	Fixed	Diotic	Right-left	0.70	1.25	2.37	0.015	**0.028**	[0.18 1.21]
Anterior	2, 3, and 4	Melodic-fixed	Monaural left	Right-left	–0.03	0.82	–0.14	0.445	0.516	[–0.36 0.31]
Anterior	2, 3, and 4	Melodic-fixed	Monaural right	Right-left	0.15	0.99	0.66	0.258	0.516	[–0.25 0.56]
Anterior	2, 3, and 4	Melodic-fixed	Diotic	Right-left	0.27	1.03	1.12	0.138	0.414	[–0.15 0.69]
Anterior	2, 3, and 4		Monaural left	Melodic-fixed (LH + RH)	0.46	0.57	3.39	0.002	**0.004**	[0.44 1.38]
Anterior	2, 3, and 4		Monaural right	Melodic-fixed (LH + RH)	0.54	0.74	3.07	0.003	**0.004**	[0.46 1.68]
Anterior	2, 3, and 4		Diotic	Melodic-fixed (LH + RH)	0.60	0.48	5.30	< 0.001	**0.001**	[0.81 1.61]
Anterior	2, 3, and 4		All	Melodic-fixed (LH + RH)	0.53	0.48	4.66	< 0.001	**0.002**	[0.33 0.73]
Posterior	2, 3, and 4		All	Melodic-fixed (LH + RH)	0.30	0.38	3.37	0.004	**0.004**	[0.15 0.46]

All contrasts refer to one-sided, pair-wise *t*-tests and target the effects of the above-specified ANOVA factors EAR OF ENTRY, HEMISPHERE (RH *minus* LH) and SEQUENCE (melodic contour *minus* fixed pitch); the region of interest (ROI) (anterior vs. posterior) and the respective notes [1st vs. (2nd, 3rd, 4th)] are also specified, together with the conditions underlying the respective comparison. The right side of the table denotes means, standard deviations (SD), *t*-values, uncorrected *p*-values and corrected *p*-values according to the Holm–Bonferroni procedure, together with the 90% confidence intervals (CI).

Regarding the neural responses to the pooled second, third and fourth note of the sequences, the respective N100m activity also included more anterior areas in auditory cortex (Figure 3B), and the response magnitude in the anterior ROI was found to differ in response to sequences with fixed vs. varying pitch. Specifically, melodic contours elicited N100m waves with larger magnitude than fixed pitch sequences, as shown by a highly significant main effect of SEQUENCE [*F*(1, 17) = 21.73, *p* < 0.001^***^]. EAR OF ENTRY was not significant as a main effect [*F*(2, 34) = 0.32, *p* = 0.725] and also not in its interaction with SEQUENCE [*F*(2, 34) = 0.50, *p* = 0.605]. Importantly, while the main effect of HEMISPHERE was highly significant [*F*(1, 17) = 11.18, *p* = 0.004^**^], its interaction with SEQUENCE was not [*F*(1, 17) = 0.60, *p* = 0.448], nor was the second-order interaction [*F*(2, 34) = 0.74, *p* = 0.455]. There was, however, an EAR OF ENTRY * HEMISPHERE interaction [*F*(2, 34) = 4.98, *p* = 0.015*], and *post hoc* tests indicated that this effect was particularly strong among the monaural conditions, whereas it was somewhat weaker (but still significant) when only diotic conditions were considered (cf. Table 1).

**FIGURE 5:**
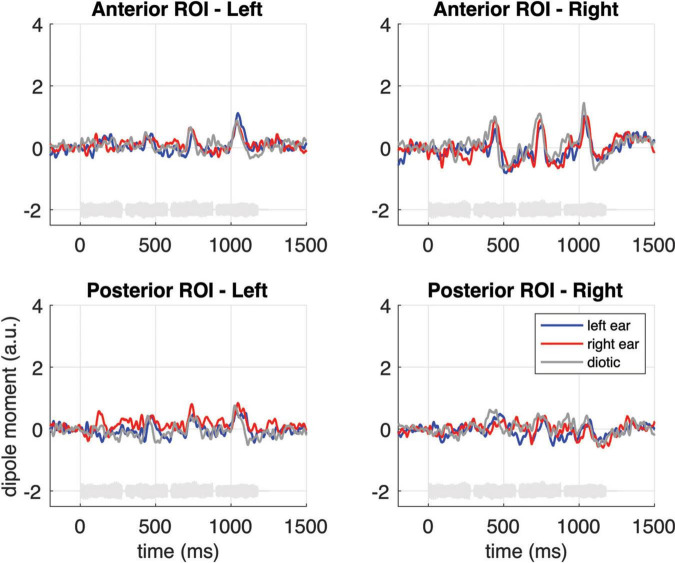
Grand-average source waveforms from the anterior (top panels) and posterior (bottom panels) ROIs, shown as difference waveforms for the contrast of melodic contours and fixed pitch sequences. The light gray curves at the bottom of each subplot represent example sound waveforms from the stimulation.

To further illustrate the neural activity associated with melodic contour processing, Figure 3C presents an MNE representation that is based on the differential activity (i.e., the contrast) between sequences with fixed vs. varying pitch. The comparison with panel B of the same figure emphasizes the activation in anterior auditory areas; correspondingly, it can be seen in Figure 5 how the contrast yields prominent difference waveforms following the second, third and fourth, but not the first note of the sequence. Notably, this pattern is clearly visible in the anterior ROI (upper panels of Figure 5), but it is greatly attenuated in the responses from the posterior ROI (lower panels of Figure 5).

As a final analysis step, we performed an additional, comprehensive repeated measures ANOVA on the pooled second, third and fourth notes in which the above-mentioned factors were supplemented with the factor ROI (posterior vs. anterior). This ANOVA was not based on a saturated model; instead, we focused on the important interaction effects that would illustrate the dissociation between the anterior and posterior activation clusters. The analysis revealed that the response difference between sequences with fixed vs. varying pitch was significant in both ROIs but strongly pronounced in the anterior ROI [ROI * SEQUENCE: *F*(1, 17) = 5.36, *p* = 0.033*; see also Figure 5 and the *post hoc* tests in the bottom panel of Table 1]; moreover, the influence of EAR OF ENTRY was larger in the posterior than in the anterior response [ROI * EAR OF ENTRY: *F*(2, 34) = 3.71, *p* = 0.039*].

## Discussion

The present study has shown how distributed MEG source modeling reveals a gradient of early melodic contour processing in human auditory cortex. Our data demonstrate that the onset of a melodic sequence is associated with pronounced neuromagnetic responses in posterior auditory areas. The subsequent notes of the sequence elicit larger responses for melodic contours as compared to fixed pitch sequences, which is predominantly reflected in the activity of anterior auditory cortex regions. The posterior onset activity is pronounced in the hemisphere contralateral to the ear of entry, whereas the response associated with melodic contour processing in anterior areas displays no hemisphere lateralization, independent of the ear of entry.

The first note of the four-note sequences evokes a large N100m wave bilaterally in posterior auditory cortex (PT; cf. Figure 3A and bottom panels in Figure 4), and we know from existing studies that this activity is dominated by the neural response to the onset of sound energy from silence (Gutschalk et al., 2002, 2004; Patterson et al., 2002; Hamilton et al., 2018). Previous work has linked the PT to the segregation and matching of spectrotemporal sound patterns (e.g., Griffiths and Warren, 2002; von Kriegstein et al., 2006; Andermann et al., 2017); moreover, posteromedial portions of the PT have been found to respond specifically to sound sequences with changing spatial positions (Warren and Griffiths, 2003; Barrett and Hall, 2006; Ahveninen et al., 2014). The latter aspect is reminiscent of our finding that diotic stimulation elicits comparable N100m magnitudes in the PT bilaterally, while monaural input leads to widespread activity that is pronounced in the contralateral hemisphere, relative to the ear of entry. This parallels earlier neuromagnetic results (Reite et al., 1982; Ackermann et al., 2001) and we speculate that it is a part of auditory feature processing at an early cortical stage, where the spatial location of the sound source is initially represented.

As the melodic contour proceeds from the first to the subsequent notes, the neuromagnetic responses in posterior parts of the auditory cortex are diminished, and the focus of activity is shifted to more anterior regions in PP (cf. Figure 3). This finding nicely resembles earlier results that were acquired by means of fMRI (Patterson et al., 2002; Barrett and Hall, 2006; Norman-Haignere et al., 2013) and is, to the best of our knowledge, reported for the first time in the MEG domain. Previously, Warren and Griffiths (2003) as well as Barrett and Hall (2006) have interpreted their results in light of a dual-streams-hypothesis (Kaas et al., 1999), and broadly speaking, the activity in our study might also follow a gradient in which melodic contour processing would be attributed to an anterior stream and spatial processing would be part of a posterior stream. Patterson et al. (2002) proposed that pitch related activity moves anterolaterally away from primary auditory cortex. The strong interconnections between anterolateral PT, lateral HG and PP (at least in the right hemisphere) as described in an *in vivo* DSI tractography study by Cammoun et al. (2015) also support this view, and histological findings in the macaque (de la Mothe et al., 2006) might be further indicative of an early segregation that is already present at the level of core auditory regions. Importantly, the anterior activity in our experiment displays a specific behavior in response to the melodic contours: Sequences with varying pitch induce much larger N100m responses than fixed pitch sequences. This difference closely matches the findings that were reported by earlier studies (Patterson et al., 2002, 2016; Andermann et al., 2021), and it is only present in PP but not in PT. Together, our data support the idea of a cortical representation for melodic contour processing which can be revealed, both in spatial and in functional terms, by applying distributed source modeling on neuromagnetic data.

There is another aspect in our data that further corroborates the idea that melodic contours are processed in more anterior auditory areas. While onset related N100m responses in posterior regions (cf. lower panels in Figures 4A–C) exhibit a strong contralateral dominance relative to the ear of entry (monaurally left or right vs. diotic), the effect is weaker (although still significant) in the anterior responses to fixed pitch sequences, and it is absent when one considers the enhancement that results from the specific contrast between fixed pitch sequences and melodic contours (cf. Figure 5). On the other hand, however, a right-hemispheric dominance in the anterior cortical response to melodic contours could not be shown in the current data. The latter aspect is somewhat surprising and it stands in contrast with the fMRI results of Patterson et al. (2002) as well as earlier investigations suggesting that pitch contour information is predominantly processed in the right temporal lobe (Zatorre, 1985; Samson and Zatorre, 1988; Griffiths et al., 1997; Liégeois-Chauvel et al., 1998; Johnsrude et al., 2000; Zatorre et al., 2002; Warrier and Zatorre, 2004). It should, however, be noted that in Patterson et al.’s (2002) study, the activation difference between hemispheres was present in some but not all listeners; moreover, neurophysiological findings also point to a more balanced processing of melodic contours between hemispheres (e.g., Fujioka et al., 2004; Andermann et al., 2021). Taken together, we conclude from our data that the neural representation of melodic contours in the anterior auditory cortex appears balanced between hemispheres.

While the current pattern of findings is in good agreement with earlier work on anterior melodic contour processing (Patterson et al., 2002; Warren and Griffiths, 2003; Barrett and Hall, 2006; Norman-Haignere et al., 2013), some cautionary note should be made with respect to the possible influence of neural adaptation. Our stimulus set included equiprobable sounds from a narrow tonal range with similar spectral composition; it therefore appears reasonable to regard the difference between sequences with fixed vs. varying pitch as mainly driven by aspects that relate to melodic contour. Nevertheless, the larger response to melodic contours might—at least to some degree—also result from a release of adaptation as pitch changes from one single note to another. Adaptive mechanisms were beyond the scope of this experiment, so it is difficult to indicate precisely to which extent adaptation has shaped the observed responses. On a conceptual level, however, it appears challenging to imagine a melodic contour where pitch does *not* vary between single notes, since the pattern of ups and downs is just what constitutes the contour (Dowling, 1978); as a consequence, one cannot access melodic properties without including pitch shifts and subsequent release of adaptation at the single note level. A way out of this “forest vs. trees” situation might be to assume that the cortical representation of melodic information actually *is* the interplay of adaptive processes integrated at multiple timescales, including but not restricted to the level of transitions between subsequent notes (in the sense of Ulanovsky et al., 2004). While such an assumption surely warrants further research, the results from our experiment provide evidence that the transient neuromagnetic activity in anterior auditory cortex poses a promising starting point for this endeavor.

A final remark should be made regarding the methodological challenges that were tackled during data analysis in our study. MNE analyses based on *l*_2_-norm calculations usually suffer from “leakage,” i.e., a certain spread of cortical representations that can be quantified by point-spread and cross-talk functions (Hauk et al., 2019). It is, however, unlikely that leakage had relevant effects on the primary target of our study, namely the auditory activation pattern in the anterior and posterior ROIs. As shown in Figure 3, the peak activity in both hemispheres clearly lies within the posterior ROI for the first note of the sequence (bottom panel of Figure 3A); in contrast, the difference between fixed pitch sequences and melodic contours (pooled second, third and fourth note) has its peak activity within the anterior ROI (bottom panel of Figure 3C). The waveforms originating from both ROIs were clearly separable (cf. Figure 4), indicating that they reflect the activity of different stages along the auditory pathway. Additional activity in adjacent areas was not considered during our analyses since it occurred outside the pre-defined ROIs and, in fact, clearly outside auditory cortex (Hackett, 2015). In sum, the use of MEG together with a finely parceled atlas appears as a valid approach to distinguish the spatio-temporal interplay of different cortical processing stages.

## Data availability statement

The datasets presented in this study can be found in online repositories. The names of the repository/repositories and accession number(s) can be found below: osf.io/zy39d.

## Ethics statement

The studies involving human participants were reviewed and approved by the Committee of the Medical Faculty, University of Heidelberg (Alte Glockengießerei 11/1, 69115 Heidelberg, Germany). The patients/participants provided their written informed consent to participate in this study.

## Author contributions

ST: data recording, formal analysis, and writing of the manuscript. MS: analysis software, formal analysis, and visualization. MA: conceptualization and writing of the manuscript. AR: conceptualization, methodology, project administration, formal analysis, and writing of the manuscript and supervision. All authors contributed to the article and approved the submitted version.
